# Upregulation of Cortical Renin and Downregulation of Medullary (Pro)Renin Receptor in Unilateral Ureteral Obstruction

**DOI:** 10.3389/fphar.2019.01314

**Published:** 2019-11-14

**Authors:** Stefanny M. Figueroa, Mauricio Lozano, Carolina Lobos, Matthew T. Hennrikus, Alexis A. Gonzalez, Cristián A. Amador

**Affiliations:** ^1^Laboratorio de Fisiopatología Renal, Instituto de Ciencias Biomédicas, Universidad Autónoma de Chile, Santiago, Chile; ^2^Instituto de Química, Pontificia Universidad Católica de Valparaíso, Valparaíso, Chile; ^3^Department of Physiology, Tulane University School of Medicine, New Orleans, LA, United States

**Keywords:** chronic kidney disease, intrarenal RAS, unilateral ureter obstruction, inflammation, pro-renin receptor

## Abstract

Chronic kidney disease (CKD) is characterized by renal dysfunction, which is a common feature of other major diseases, such as hypertension and diabetes. Unilateral ureteral obstruction (UUO) has been used as a model of CKD in experimental animals and consists of total obstruction of one kidney ureter. The UUO decreases renal blood flow, which promotes the synthesis of renin in the juxtaglomerular apparatus, the first step in renin–angiotensin system (RAS) cascade. RAS induces inflammation and remodeling, along with reduced renal function. However, it remains unknown whether intrarenal RAS (iRAS) is activated in early stages of CKD. Our objective was to characterize different iRAS components in the renal cortex and in the medulla in an early phase of UUO. Male C57BL/6 mice (8–12 weeks old) were subjected to UUO in the left kidney, or to sham surgery, and were euthanized after 7 days (*n* = 5/group). Renal function, renal inflammatory/remodeling processes, and iRAS expression were evaluated. UUO increased plasma creatinine, right renal hypertrophy (9.08 ± 0.31, *P* < 0.05 vs. Sham), and tubular dilatation in the left kidney cortex (42.42 ± 8.19µm, *P* < 0.05 vs. Sham). This correlated with the increased mRNA of IL-1β (1.73 ± 0.14, *P* < 0.01 vs. Sham, a pro-inflammatory cytokine) and TGF-β1 (1.76 ± 0.10, *P* < 0.001 vs. Sham, a pro-fibrotic marker). In the renal cortex of the left kidney, UUO increased the mRNA and protein levels of renin (in 35% and 28%, respectively, P < 0.05 vs. Sham). UUO decreased mRNA and protein levels for the (pro)renin receptor in the renal medulla (0.67 ± 0.036 and 0.88 ± 0.028, respectively, *P* < 0.05 vs. Sham). Our results suggest that modulation of iRAS components depends on renal localization and occurs in parallel with remodeling and pro-inflammatory/pro-fibrotic mechanisms.

## Introduction

Chronic kidney disease (CKD) is a worldwide health problem closely linked to other major diseases (Tonelli et al., 2015) that is difficult to diagnose and affects around 10% of the population (De Nicola and Zoccali 2016). CKD increases cardiovascular mortality and is a risk multiplier for patients with hypertension and/or diabetes mellitus (Couser et al., 2011). CKD is defined by a sustained reduction in glomerular filtration rate (GFR) (Levey et al., 2011), which involves structural or functional anomalies of the kidneys evidenced on urine analysis (proteinuria) (Tonelli et al., 2011), biopsy, or imaging. Evidential structural abnormalities in CKD are characterized by glomerulosclerosis, tubular atrophy, and tubulo-interstitial fibrosis (Menon and Ross 2016), three conditions that drive nephrons to deterioration and to irreversible renal failure.

The unilateral ureteral obstruction (UUO) is a physiologically relevant *in vivo* model of renal inflammation that leads to fibrosis (Yang et al., 2010). UUO recapitulates the fundamental pathophysiologic mechanisms that typify all forms of human CKD in a relatively short time span (Eddy et al., 2012). It has been demonstrated that UUO increases interstitial capillary permeability (Yamaguchi et al., 2012) and the levels of IL-1-linked cytokines and transforming growth factor-β1 (TGF-β1), which correlate with the recruitment of inflammatory mononuclear cells (Eddy and Neilson 2006; Chi et al., 2017). Cao et al., 2015 described that during the first week, this pro-inflammatory phenotype is crucial for renal fibrosis consolidation observed after 4 weeks of UUO in mice (Cao et al., 2015). Thus, the UUO surgery in rodents can be used as an experimental model for studying the early (inflammatory, 7 days) (Ucero et al., 2014) and/or consolidated (fibrotic, 14 days) stage of CKD (Martínez-Klimova et al., 2019).

Ureteral constriction reduces the GFR and induces a renin release (Eide et al., 1977), which represent the first step of renin–angiotensin system (RAS) activation (Kobori et al., 2007). This effect is considered to be critical because kidneys possess an intrinsic RAS (or intrarenal RAS, iRAS) that can also regulate the water and sodium balance and blood pressure (Velez 2009). In this context, it has been demonstrated that 24 h of UUO downregulates the mRNA levels of renal angiotensin-II (AngII) type 1 receptor (AT_1_R) (Pimentel et al.,1994) and increases the mRNA levels of renin from the juxtaglomerular apparatus (Pimentel et al., 1993). These changes, of which most were characterized 20 years ago, suggest that obstructive uropathy rapidly leads to hemodynamic alterations that may be implicated in the onset of CKD. Renin is also expressed in the collecting duct and is secreted by the principal cells in the renal medulla. Renin binds to the (pro)renin receptor (PRR, named also ATPase, H^+^ transporting lysosomal accessory protein 2—*Atp6ap2*) (Nehme et al., 2019) at the neighboring intercalated cells (also in the medulla), thus activating pro-renin in a catalytic or non-catalytic form and increasing intratubular AngII formation while also triggering intracellular cascades related to fibrosis (Gonzalez et al., 2017).

We hypothesized that iRAS activation occurs during the early structural changes and the pro-inflammatory phase induced by UUO. This would be crucial for fibrosis consolidation and renal remodeling. Therefore, the main objective of our work was to characterize the iRAS components such as renin and PRR in the renal cortex and in the medulla at 7 days of UUO in mice.

## Materials and Methods

### Animals and Experimental Protocol

For UUO, 9- to 12-week-old male C57BL/6 mice (*n* = 5) were anesthetized with a solution of ketamine/xylazine (80:8 mg kg^−1^) and the left ureter was exposed by a dorsal incision. Then the ureter was obstructed by two-point ligation with silk sutures. Sham-operated mice (*n* = 5) underwent the same procedure, except for the obstruction of the left ureter (Ucero et al., 2014). All the experimental procedures with animals were performed according to the Committee on Animal Research and Ethics (Guidelines for Ethical Conduct in the Care and Use of Nonhuman Animals in Research, 2012) and were under the surveillance of the Ethics Committee of the Universidad Autonoma de Chile. Animals were placed in conditions of light–dark cycle (12 h), temperature of 21°C, humidity of 50%, adequate ventilation, noise-free, food and water *ad libitum*, and under continuous veterinary monitoring.

For histological and biochemical analyses, the animals were sacrificed 7 days after surgeries and the kidneys were quickly removed through a dorsal incision. Plasma was collected and stored at −20°C. Plasma creatinine and blood urine nitrogen (BUN) were analyzed using automated chemistry Jaffe assay and UV urease kit, respectively (Valtek Diagnostics, Santiago, Chile).

### Histological Analysis

Sagittal sections of obstructed kidneys were fixed in Bouin (Sigma Aldrich, USA), embedded in parafﬁn, and 5-µm sections were prepared using a Leica RM2235 microtome (Leica Microsystems, Shanghai). The sections were washed and stained with hematoxylin–eosin (Sigma Aldrich, USA) for the assessment of tubular diameter. Images for cortices and medullas were captured with a Nikon digital sight DS-U3 digital camera, attached to a Nikon Upright Microscope ECLIPSE Ci-L (Nikon Instruments Inc., USA), and analyzed using ImageJ 1.43u software (NIH, USA).

### Total RNA Isolation and qrt-PCR

Total RNA from whole kidney, cortex, and medulla was extracted with the TRIzol reagent, according to the manufacturer’s instructions (ThermoFisher Scientific, USA). For whole kidney and renal cortex, cDNA was synthesized from 2 µg of RNA using M-MLV reverse transcriptase kit^®^ (Promega, USA), while in the medulla, cDNA was synthesized from 1 µg of RNA using Superscript II reverse transcriptase kit^®^ (ThermoFisher Scientific, USA). Real-time PCR reactions were performed in a LightCycler96^®^ (Roche, Switzerland), and transcript levels were detected by SYBR Green method using FastStart Essential DNA Master mix (Roche, Switzerland). The following primers were used: 18S: (F) 5′-GCC GCTAGAGGTGAAATTCTTGGA-3′, (R) 5′-ATCGCCAGTGGCATCGTTTAT-3′; angiotensin-converting enzyme (ACE)1: (F) 5′-ATGAAGCCAAGGCTGACAGGTT-3′, (R) 5′-GTGATTGGAT ACCTCCGTTGCTT-3′; ACE2: (F) 5′-GGGGTGAAATTCCCAAAGAGCAGT-3′, (R) 5′-TCTGC CCAGCTTCAGTGGAATTTG-3′; AGT: (F) 5′-ACTGCTCC​AGGCTTTCGTCTAA-3′, (R) 5′-TC CACCCTGTCACAGCCT​TTAT-3′; AT1Ra: (F) 5′-ACACTGCCATGCCCATA​ACCA-3′, (R) 5′-T AGACAGGCTTGAGTGCGACTT-3′; IL-1β: (F) 5′-ACTGGTACATCAGCACCTCACAAG-3′, (R) 5′-GCT​GTCTGCTCATTCACGAAAAGG-3′; PRR (*Atp6ap2* gene): (F) 5′-GCGGGTGCTTTA GGGAATGAAT-3′, (R) 5′-TGG​CCAAGACAGGTCTTCCTTT-3′; REN1: (F) 5′-AGCTCCCT​GA AGTTGATCATGC-3′, (R) 5′-CTCCTGTTGGGATACTGT​AGCA-3′; TGF-β1: (F) 5′-TACGCCT GAGTGGCTGTC​TTTT-3′, (R) 5′-TTGGGGCTGATCCCGTTGATTT-3′. All PCR products were subjected to a melting curve program to confirm amplification specificity. Results were analyzed according to the standard curve method and mRNA level was calculated with respect to the relative amount of 18S for each sample.

### Protein Analysis by Western Blot

For protein extraction, a lysis buffer containing 50 mM Tris-HCl (pH 7.5), 0.1 mM NaCl, 0.5% Triton X-100, 0.5 M EDTA, and a mixture of protease/phosphatase inhibitors (Roche, Switzerland) were used. We used a D-160 Scilogex polytron (ABC Scientific, USA) and sonication at 130 W, 20 KHz and 60% amplification (Ultrasonic Processor, USA) to homogenize the tissues. After centrifugation at 7,500×*g* for 5 min at 4°C, the supernatant was collected and total protein concentration in the sample was measured using a BCA protein assay kit (ThermoFisher Scientific, USA). Samples containing equal amounts of protein (40 µg for cortex and 10 µg for medulla/lane) were separated based on their molecular weight by 7.5% polyacrylamide gels for electrophoresis (SDS-PAGE) and transferred onto nitrocellulose membranes at a constant current of 2.5 A, 25 V for 7 min (BioRad, USA). After blocking with 5% non-fat milk in Tris-buffered saline with 0.1% Tween-20 (TBST) for 1 h at room temperature (RT), the membranes were stain with ponceau red and later incubated with primary antibodies against renin and ACE (both used at 1:200; Santa Cruz Biotechnology, USA), angiotensinogen (AGT) (1:200, Immuno-Biological Laboratory, Japan), PRR (1:250, Sigma Aldrich, USA), and α-tubulin (1:40,000, Sigma Aldrich, USA) overnight at 4°C. Subsequently, the membranes were washed with TBST and incubated with the corresponding secondary antibodies (including goat anti-mouse IgG and mouse anti-rabbit IgG, 1:3,000) for 1 h at RT, and finally visualized by enhanced chemiluminescence reagents (ThermoFisher Scientific, USA). The relative expression level of proteins was analyzed using ImageJ software.

### Statistics

Results from *in vivo* studies were expressed as the mean ± SEM. Data were analyzed by one-way analysis of variance (ANOVA) test followed by Bonferroni posttest (more than two groups), by Student’s *t* test, or Mann–Whitney nonparametric test (two groups), as appropriate. All analyses were performed using GraphPad Prism (GraphPad Software, USA). Values of *P* < 0.05 were considered as statistically significant.

## Results

### UUO Induces an Increase in Plasma Creatinine and Hypertrophy in Contralateral Kidney

After 7 days of total left ureteral obstruction, mice presented with right kidney hypertrophy, as shown by the increase in right kidney weight/tibia length ratio (9.76 ± 0.42 g, *P* < 0.05; **Table 1**). No difference was detected in left kidney weight.

**Table 1:** UUO induces a contralateral renal hypertrophy and a rise of plasma creatinine.

Group	Sham	UUO
Total weight (g)	23.77 ± 0.45	22.28 ± 0.61
Left kidney weight/tibia length (mg/mm)	8.09 ± 0.35	8.41 ± 0.38
Right kidney weight/tibia length (mg/mm)	8.13 ± 0.26	9.76 ± 0.42*
Blood urea nitrogen (mg/dl)	58.32 ± 1.87	59.19 ± 7.49
Creatinine (mg/dl)	0.368 ± 0.021	0.574 ± 0.117*

Physiological parameters at 7 days of UUO BUN blood urea nitrogen, UUO unilateral ureteral obstruction Mann–Whitney statistic test. *P < 0.05 vs. Sham group (n = 5).

As an indirect way to determine GFR reduction in our model, we observed that UUO induced an increase in plasma creatinine in relation to the Sham group (0.57 ± 0.12 mg/dl, *P* < 0.05), but did not increase the BUN.

### UUO Produces a Pro-Inflammatory and Pro-Fibrotic Status in the Obstructed Kidney

**Figure 1:**
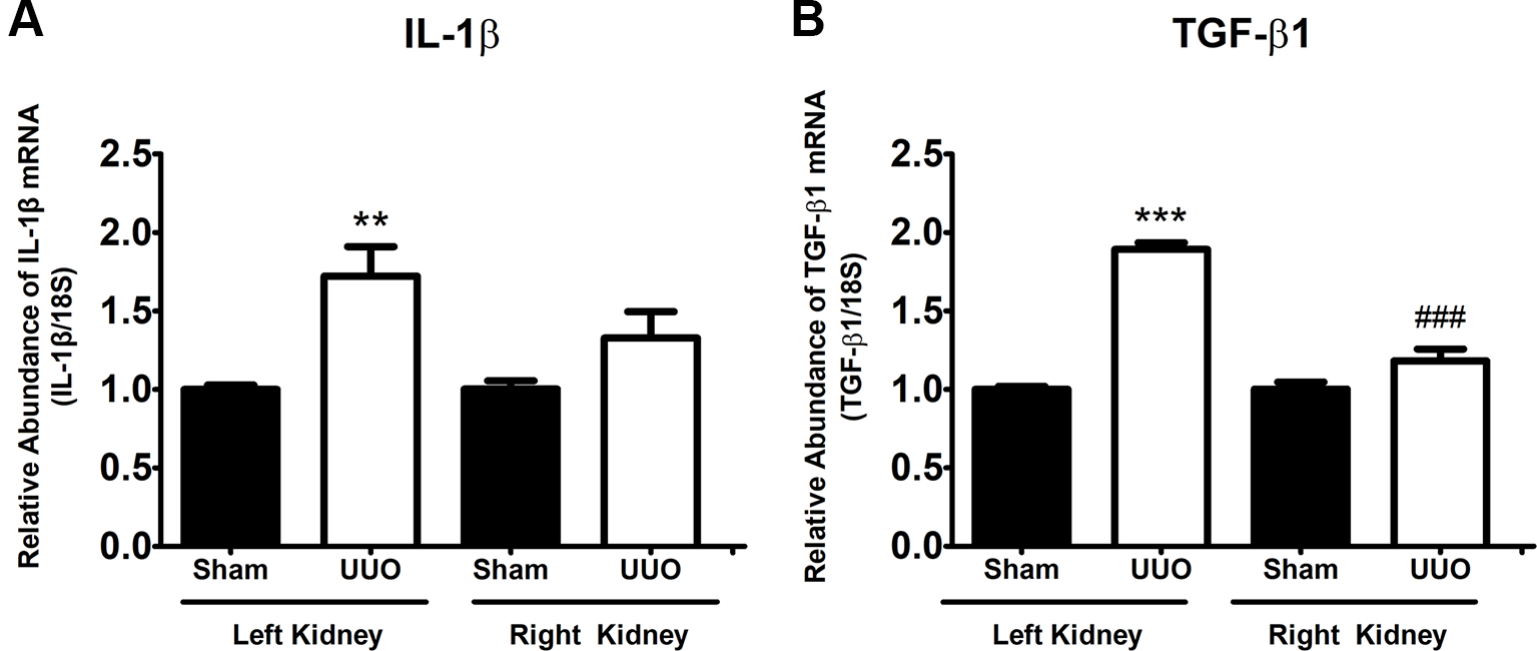
Unilateral ureteral obstruction (UUO) induces a pro-inflammatory and pro-fibrotic phenotype at 7 days. **(A)** IL-1β and **(B)** TGF-β1 mRNA levels in the left and right kidneys. 18S mRNA was used as the housekeeping gene. One-way ANOVA test followed by Bonferroni test was performed. **P 0.01 and ***P 0.001 vs. Sham group. ^###^ *P* 0.001 vs. UUO left kidney group (*n* = 5).

Since our aim was to study the UUO during the pro-inflammatory phase leading to the pro-fibrotic phenotype, we determined the levels of interleukin-1β (IL-1β) and TGF-β1, as a major pro-inflammatory cytokine involved in tubulointerstitial injury (Tesch et al., 1997) and as the main fibrosis-promoting factor (Ding et al., 2014), respectively. The analysis by quantitative RT-PCR demonstrated that IL-1β (1.7-fold change, FC, vs. Sham, *P* < 0.01) and TGF-β1 (1.9 FC vs. Sham, *P* < 0.001) were increased in the left (obstructed) kidney after 7 days of UUO (**Figures 1A, B**, respectively). These effects were not observed at level of the right kidney and were in concordance with previous results reported (Pimentel et al., 1995;Wang et al., 2015).

### UUO Induces Tubular Remodeling in Cortex and Medulla of Obstructed Kidney

**Figure 2:**
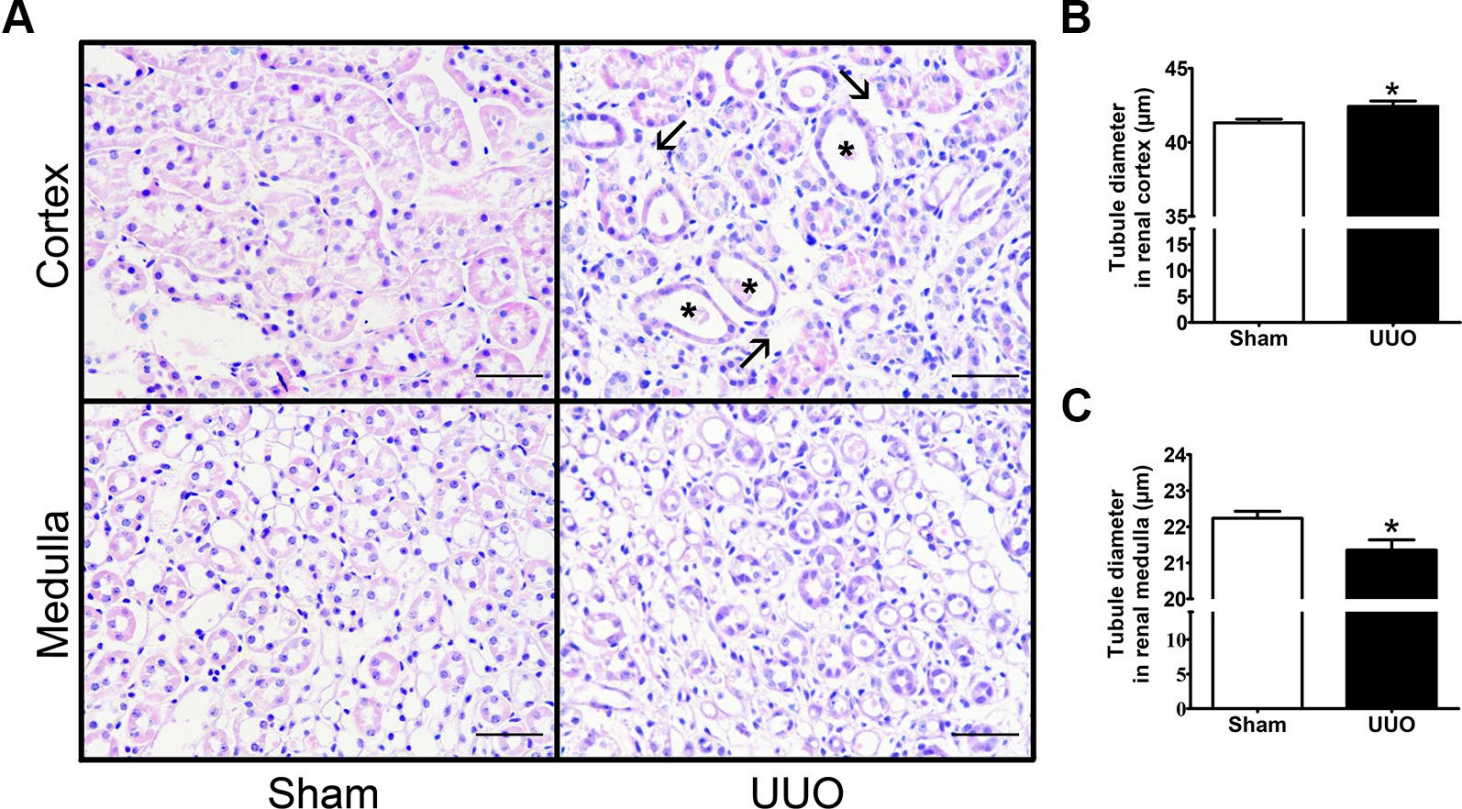
Unilateral ureteral obstruction (UUO) induces tubular remodeling in the renal cortex and medulla after 7 days. **(A)** Haematoxylin–eosin staining in the renal cortex and medulla (*scale bar*, 100 µm). *Black arrows* show the increase of tubule interstitial space, while *black asterisks* show tubular dilation. Quantification of tubule diameter in the **(B)** cortex and **(C)** medulla of Sham and UUO groups. Mann–Whitney statistic test. *P 0.05 vs. Sham group (*n* = 5).

In order to explore the tubular remodeling induced by UUO in the renal cortex and in the medulla, we analyzed by light microscopy transversal sections of the left kidney stained by hematoxylin–eosin (**Figure 2**). We observed that UUO induced an increase of tubule interstitial space (**Figure 2**, black arrows) and an enlargement of diameter in cortical tubules (**Figure 2**), which has been related with the increase of the tubular lumen area in obstructive uropathy (*P* < 0.05) (Cavaglieri et al., 2015). On the other hand, we detected a diminution in tubular diameter of kidney medulla with ureteral obstruction (*P* < 0.05) (**Figure 2**), suggesting a differential remodeling of kidney at 7 days of UUO.

### UUO Induces Differential Changes of iRAS Components in Left Renal Cortex and Medulla at mRNa Level, but not at Protein Level

**Figure 3:**
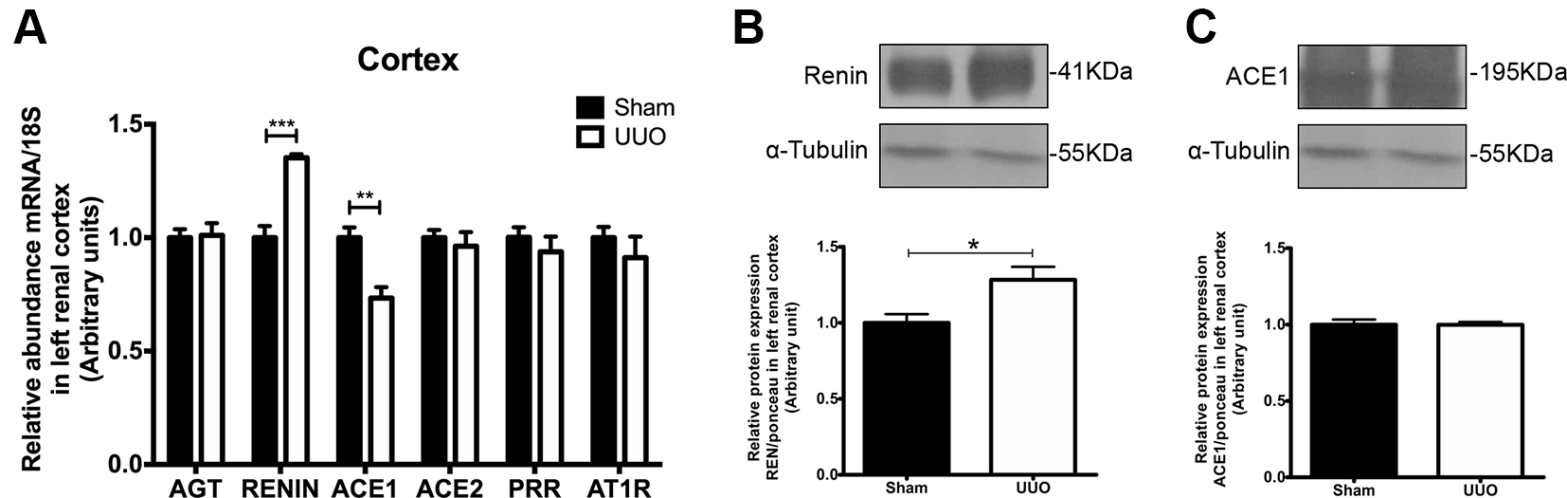
Unilateral ureteral obstruction (UUO) induces upregulation of renin in left renal cortex. **(A)** mRNA levels for: angiotensinogen (AGT), renin, angiotensin-converting enzyme (ACE)1/2, (pro)renin receptor (PRR), and angiotensin-II receptor type 1 (AT1R) in the renal cortex. 18S mRNA was used as the housekeeping gene in qRT-PCR studies. Western blot and immunodetection of **(B)** renin and **(C)** ACE1 in the renal cortex. α-tubulin was used as internal loading control. Student’s *t* test was performed. **P < 0.01 or ***P < 0.001 vs. Sham group (*n* = 5).

As the renal remodeling induced by 7 days of UUO was not equal in the renal cortex and in the medulla, we studied the levels of iRAS components separately. In the renal cortex of the left kidney, UUO promoted an induction of renin (1.4 FC vs. Sham, *P* < 0.001) and a reduction of ACE1 mRNA (0.3 FC vs. Sham, *P* < 0.01) (**Figure 3A**), suggesting a differential response of iRAS. Only the renin increase was confirmed at protein level (**Figure 3B**). No differences were detected for the mRNA levels of AGT, ACE2, PRR, or AT1R in the renal cortex (**Figure 3A**), and neither for ACE1 at protein level (**Figure 3C**).

**Figure 4:**
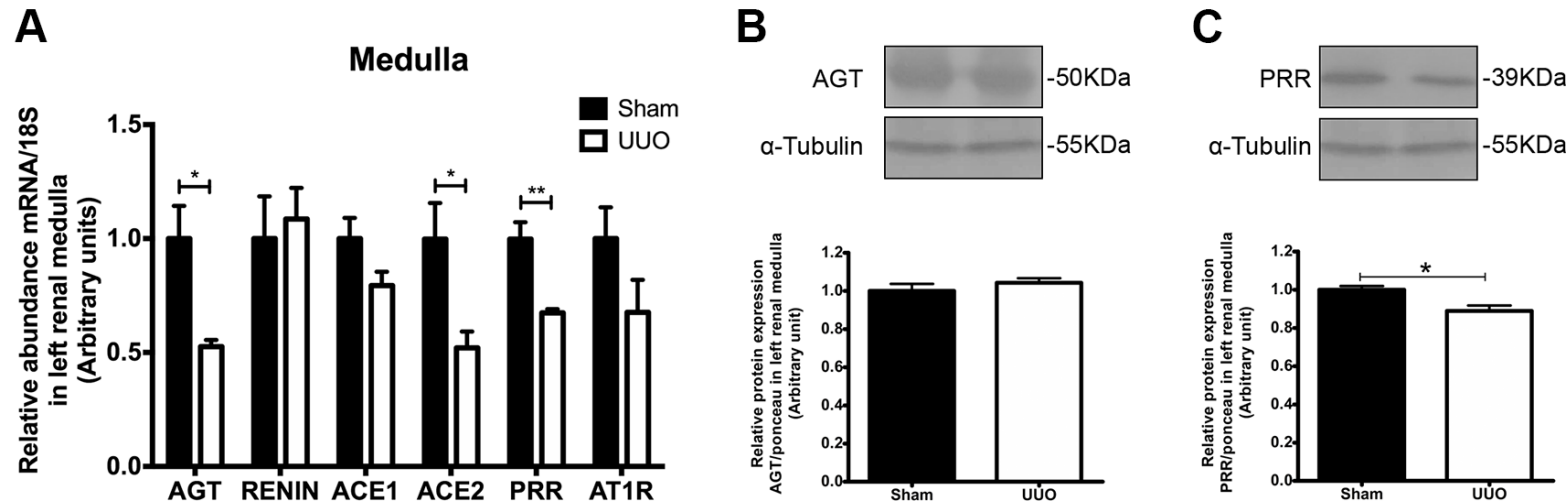
UUO induces downregulation of (pro)renin receptor (PRR) in left renal medulla. **(A)** mRNA levels for: AGT, renin, ACE1/2, PRR, and AT1R in renal medulla. 18S mRNA was used as the housekeeping gene in qRT-PCR studies. Western blot and immunodetection of **(B)** AGT and **(C)** PRR in renal medulla. α-tubulin was used as internal loading control. Student’s *t* test was performed. *P < 0.05 or **P < 0.01 vs. Sham group (*n* = 5).

On the other hand, UUO diminished significantly the mRNA level for AGT, for ACE2 (in both cases 0.5 FC vs. Sham, *P* < 0.05), and for PRR (0.3 FC vs. Sham, *P* < 0.01) in renal medulla of the left kidney (**Figure 4A**). In the case of protein expression, we corroborated the diminution only for PRR in renal medulla after UUO (**Figures 4B, C**). No differences were detected in medullary renin or ACE1 (*P* = 0.09). In the case of AT1R, we observed a modest reduction of mRNA (**Figure 4A**), which has been reported previously by Pimentel et al. at 24 h of UUO and by non-quantitative techniques (Pimentel et al.,1994).

## Discussion

Different mediators belonging to the RAS have been described in renal tissue for more than 30 years where their activation may promote or accelerate CKD. For instance, it has been demonstrated that in diabetes mellitus and hypertension, the two major risk factors of CKD, the urinary AGT (Hollenberg et al., 2003) and the intrarenal AngII (Navar et al., 2011), are induced. Here, by using an experimental CKD model that promotes renal inflammation driving to fibrosis, we have characterized different elements of iRAS in the cortex and in renal medulla.

**Figure 5:**
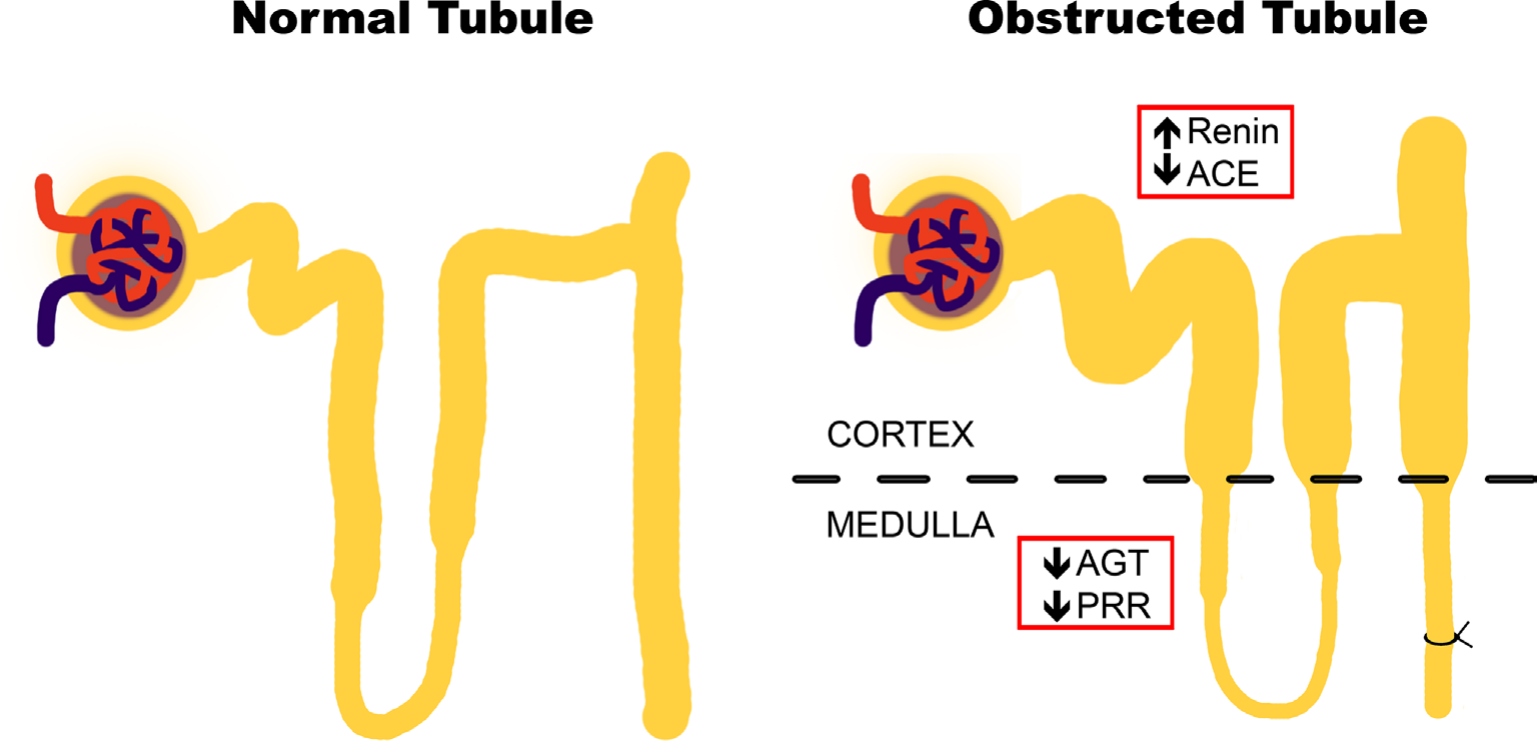
Proposal of changes in intrarenal renin–angiotensin system (iRAS) components during the inflammatory stage of UUO. Obstructive uropathy at 7 days (recognized as “the pro-inflammatory phase”) induces tubular remodeling and iRAS activation in the cortex, as reflected by the renin increase in dilated tubules. This activation may be counteracted by the iRAS components in the medulla, particularly by PRR.

Our main findings suggested that during the pro-inflammatory phase of obstructive uropathy, where the tubular remodeling is also observed, the iRAS is activated in the cortex as reflected by the increase in renin in dilated proximal tubules (**Figure 5**). This observation is in accordance with studies from Eide and collaborators, which demonstrated that ureteral constriction in dogs induces rapid renin release (Eide et al., 1977). In this study, renin upregulation was observed at mRNA and protein levels specifically in the cortex of obstructed kidneys after 7 days (**Figure 3**). An additional way for confirming an “iRAS activation” is the measurement of AngII; it has been demonstrated that 15 h of UUO in dogs increases the plasma AngII (Frøkiaer et al., 1992). However, this has not been demonstrated directly in kidney tubules of mice with ureteral obstruction due to the amount of tubular fluid necessary for AngII analysis. Alternatively, the increase of urinary AngII has been used as a marker of iRAS activation during CKD development in rats (Fang et al., 2018). In general, this iRAS activation results in hemodynamic changes with GFR reduction, observed as a plasma creatinine increase in UUO mice and in increases in the intraluminal pressure leading to tubular dilation observed in the proximal tubule (**Table 1** and **Figure 2**, respectively).

Since renin and pro-renin are synthetized by the collecting ducts cells in kidneys, it has been hypothesized that AngII production has a crucial role in renal compartments and a local biological activity (Nehme et al., 2019). The experimental evidence suggests that pro-renin and renin generation is critical for intratubular AngII formation and for interaction with PRR, promoting tubular damage and further intratubular AngII formation (Gonzalez et al., 2017). In this context, it is proposed that PRR activation contributes directly to the generation of some pro-inflammatory mediators (for instance, prostaglandin E2), as an additional mechanism for renal damage and remodeling (Carlsen et al., 2010; Gonzalez and Prieto 2015). Since renin is increased during the pro-inflammatory phase of UUO at the cortical level, we propose that the downregulation of medullary PRR, corroborated at mRNA and protein levels (**Figure 4A**), may act as a negative feedback of the axis. This represents an integrative iRAS modulation between the cortex and medulla (**Figure 5**).

Cumulative evidence supports the beneficial effects of direct renin antagonism in UUO models. It has been demonstrated that aliskiren prevents the renal aquaporin-2 downregulation (Wang et al., 2015) and prevents the interstitial fibrosis and collagen accumulation at renal level induced by 7 days of UUO (Chung et al., 2017). In addition, it has been reported that the anti-inflammatory and anti-fibrotic effects observed by aliskiren can be achieved alone or in combination with calcium channel blockers and valsartan (Wu et al., 2010). On the other hand, Fang and collaborators showed that the administration of PRO20, a decoy inhibitor of PRR, prevents renal increase of TGF-β1, oxidative stress, and interstitial fibrosis in rats with albumin overload (Fang et al., 2018). They demonstrated that PRR antagonism dramatically reduces the proteinuria, which has been recognized as an independent risk factor of CKD progression. This has not been demonstrated with other renin receptors, such as the renin-binding protein (RnBP) nor the mannose 6-phosphate/insulin-like grow factor II (M6P/IGFII) receptor (Batenburg and Danser 2012). Recently, Nehme et al. have highlighted that the regulation of PRR and other mediators of non-classical systemic RAS, such as ACE2 (called “extended RAS”, and also evaluated by us in **Figures 3** and **4**) (Nehme et al., 2015; Nehme et al., 2016), can be used for the development of more effective and selective pharmaceuticals therapies.

In summary, we demonstrate that the pro-inflammatory phase of UUO involves tubular damage, with cortical upregulation of renin (juxtaglomerular) and medullary PRR downregulation. This scenario suggests a differential iRAS modulation as part of the mechanisms involved in the early stages of CKD.

## Data Availability Statement

The raw data supporting the conclusions of this manuscript will be made available by the authors, without undue reservation, to any qualified researcher.

## Ethics Statement

The animal study was reviewed and approved by Ethic Commitee of the Universidad Autónoma de Chile.

## Author Contributions

CA and AG designed the experiments. SF conducted most of the experiments and collected and analyzed the data. ML and CL conducted specific experiments and collected and analyzed their respective data. CA and SF wrote the manuscript, and AG contributed to its revision. MH wrote and revised the manuscript.

## Funding

This work was supported by FONDECYT Iniciación #11150542 and DIUA157-2019 project from Vicerrectoría de Investigación y Postgrado (VRIP) (CA). In addition, SF received support from the scholarships for undergraduate (VRIEA-Pontificia Universidad Católica de Valparaíso, #039.407/2017) and PhD students (VRIP-UA).

## Conflict of Interest

The authors declare that the research was conducted in the absence of any commercial or financial relationships that could be construed as a potential conflict of interest.
